# Microbiological Risks of Traditional Raw Cow’s Milk Cheese (Koryciński Cheeses)

**DOI:** 10.3390/foods13091364

**Published:** 2024-04-28

**Authors:** Aleksandra Antoszewska, Elżbieta Maćkiw, Joanna Kowalska, Małgorzata Patoleta, Maja Ławrynowicz-Paciorek, Jacek Postupolski

**Affiliations:** Department of Food Safety, National Institute of Public Health NIH-National Research Institute, 24 Chocimska Str., 00-791 Warsaw, Poland; aantoszewska@pzh.gov.pl (A.A.); jkowalska@pzh.gov.pl (J.K.); mlawrynowicz-paciorek@pzh.gov.pl (M.Ł.-P.); jpostupolski@pzh.gov.pl (J.P.)

**Keywords:** Koryciński cheeses, pathogenic bacteria, raw milk, food safety

## Abstract

Traditional and regional foods have been increasing in popularity among consumers in Poland for many years. The observed trend of searching for natural and authentic taste encourages many producers to craft products from raw milk, including Koryciński cheeses. The aim of this study was to assess the microbiological hazards resulting from the presence of pathogenic bacteria in Koryciński cheeses available in retail trade. The tests were carried out using accredited methods, including the detection of the presence of *Salmonella* spp., the enumeration of *Listeria monocytogenes*, the enumeration of coagulase-positive staphylococci, and the detection of staphylococcal enterotoxins in food when the number of coagulase-positive staphylococci in the sample exceeded the limit of 10^5^ cfu/g. The research material consisted of 45 Koryciński cheeses. The tests conducted revealed that *Salmonella* spp. was not detected in any of the examined cheeses. However, coagulase-positive staphylococci were present in 68.9% of the samples. In as many as 15 tested cheeses, the level of *S. aureus* contamination was above 10^5^ cfu/g; therefore, these samples were tested for the presence of staphylococcal enterotoxins. The presence of staphylococcal enterotoxins was found in one Koryciński cheese. In four cheeses, the number of *L. monocytogenes* exceeded the level of 10^2^ cfu/g, the limit specified in Regulation 2073/2005 on microbiological criteria for foodstuffs. The obtained research results confirm the validity of monitoring the microbiological quality of Koryciński cheeses and the need to increase awareness of ensuring proper hygienic conditions of production, including the increased risk associated with unpasteurized milk products.

## 1. Introduction

Traditional and regional foods have noted increasing popularity among consumers in Poland for many years. These products are often associated with healthiness and unique flavors. The observed trend of searching for natural and authentic taste encourages many producers to make artisanal products from raw milk, including Koryciński cheeses [1].

Cheese is one of the more diverse groups of dairy products in terms of chemical composition and organoleptic properties. This diversity is due to both the quality of ingredients and the production process [2]. Koryciński homemade cheese (*ser koryciński swojski*, PGI) was entered in the register of protected designations of origin and protected geographical indications in Commission Implementing Regulation (EU) No 728/2012 of 7 August 2012. Protected geographical indications (PGI) are a European mark awarded to regional products of exceptional quality, with a name referring to the place where they are produced and emphasizing their link to that place [3,4].

Koryciński cheese is a soft, short-ripened cheese made from raw, whole cow’s milk with the addition of rennet and salt. It should be characterized by the shape of a flattened ball, up to 30 cm in diameter, weighing between 2.5 kg and 5 kg, and having numerous small holes of different sizes and shapes. While spices and herbs may be added for flavor enhancement, they must not alter the cheese’s fundamental characteristics. Raw milk can be filtered to remove macroscopic impurities and then cooled to ambient temperature. Cheese production should take place no later than 5 h after milking. The production uses unpasteurized cow’s milk, which is coagulated with rennet at a temperature of 33–36 °C. Koryciński cheese ripening depends on the individual production conditions and lasts from 2 to 6 weeks at room temperature, which leads to significant differences in the sensory characteristics of the cheeses made by different producers [5].

Cheese microbiological safety is mainly influenced by the microbiological quality of the raw milk and additives (spices and herbs), as well as the parameters of the individual stages of the process. Good hygiene practices are required in the making of dairy products. These are outlined in, among others, the European Guide for Good Hygiene Practices in the production of artisanal cheese and dairy products [6]. It is also important to ensure the traceability of the product through the possibility of determining the origin of all the ingredients used in its production.

Microbiological hazards in food are recorded in the Rapid Alert System for Food and Feed (RASFF). In 2021–2022, a total of 89 notifications regarding the presence of pathogens in cheese were reported to the RASFF system. Among these notifications, the majority were related to the occurrence of *Listeria* (61 notifications), including 53 of *L. monocytogenes*. This was followed by the presence of *E. coli* (20 notifications), including 16 of STEC. Additionally, there were eight notifications related to the detection of *Salmonella* spp. in cheese. During this time, two notifications concerning cheeses from Poland were recorded in this system. Both notifications addressed the presence of *L. monocytogenes* in the cheese. However, these were not Koryciński cheeses but mozzarella and Edam.

Cheese, including Koryciński cheese, is a source of valuable nutrients but also provides an ideal environment for the proliferation of microorganisms, including pathogenic bacteria. About 70% of foodborne outbreaks related to cheese consumption are caused by products derived from unpasteurized milk [7].

When it comes to cheeses made from raw cow’s milk, risks are posed by pathogens such as *Salmonella* spp., *Listeria monocytogenes*, or *Staphylococcus aureus* and the toxins they produce (staphylococcal enterotoxins).

According to the food safety criteria laid down in Commission Regulation (EC) No. 2073/2005, cheeses, butter, and cream produced from raw milk or from milk treated at a temperature lower than pasteurization temperature shall be free from the presence of *Salmonella* spp. in 25 g. In ready-to-eat foods placed on the market during their shelf life, where *L. monocytogenes* growth is possible, other than food intended for infants or food for special medical purposes, including, for example, in cheeses, the *L. monocytogenes* count should not exceed 100 cfu/g. However, for RTE products that have not left the immediate control of the producer, the quality is considered satisfactory if all five samples indicate the absence of *L. monocytogenes*. However, in terms of the process hygiene criteria, limits on the number of coagulase-positive staphylococci are set for cheeses made from raw milk. According to the regulation, the quality of these products is satisfactory when all the test results are less than 10^4^ cfu/g. On the other hand, the quality is acceptable when the test results for two out of five samples tested are between 10^4^ and 10^5^ cfu/g, while the other three values are ≤10^4^ cfu/g. For cheeses made from milk treated at a temperature lower than pasteurization temperature and for ripened cheeses made from milk or whey, which have been pasteurized or treated at a higher temperature, the product quality is satisfactory if all test results are less than 10^2^ cfu/g. In contrast, the quality is acceptable when the test results for two out of five samples tested are within the range between 10^2^ and 10^3^ cfu/g, while the other three values are ≤10^2^ cfu/g. The criteria set are aimed at improving the hygiene of production and the selection of ingredients. According to Regulation 2073/2005, when the count of coagulase-positive staphylococci, following the production process of selected food products, such as cheese, exceeds 10^5^ cfu/g, it is mandatory to test for the presence of staphylococcal enterotoxins [8].

The majority of bacterial poisonings and foodborne infections associated with the consumption of contaminated food in Poland are caused by *Salmonella* spp. [9]. These bacteria reside mainly in the digestive tracts of mammals, birds, and diverse food products [10]. *Salmonella* spp. cause the infectious disease of humans and animals—salmonellosis [11]. According to the “One Health” report published by EFSA and ECDC, salmonellosis was the second most common zoonotic disease after campylobacteriosis in the EU in 2021 [12]. Salmonellosis most commonly manifests as gastroenteritis with a variety of symptoms. Rarely, it can also develop into a systemic infection with a severe course. These bacteria can grow at a wide temperature range from 2 to 50 °C, with optimum growth at 35–37 °C, and pH 4.0–9.5, with optimum pH 6.5–7.5. Good conditions for the survival and growth of *Salmonella* spp. cells are found in environments with water activity a_w_ > 0.93, but they can also survive in food with low a_w_ [13]. Water activity in cheese ranges from 0.97 to 0.98; therefore, cheese is a good environment for the growth of these microorganisms.

*L. monocytogenes* is a species of pathogenic bacteria occurring widely in nature; they are found in soil, water, and on the surface of plants, but also in various food products (e.g., RTE, including cheese) [14,15]. The *L. monocytogenes* cause a disease—listeriosis, which has a remarkably high mortality rate of up to 30% when neurological symptoms occur. Listeriosis is particularly dangerous for people with weakened immune systems, the elderly, pregnant women, newborns, and children [16]. The *L. monocytogenes* bacteria exhibit the ability to grow at a wide temperature range from 0 to 50 °C, including refrigeration temperatures [15,17]. They are also characterized by long-term survival in fatty foods, such as cheese [17]. These bacteria can also adhere and grow as biofilm, colonizing the surfaces of various materials used in the food industry. *L. monocytogenes* tend reside in both humid and cool places, e.g., on the surface of equipment and production lines, but also on dry surfaces, i.e., cheese ripening mats, posing the risk of secondary or cross-contamination. In addition, biofilms formed by these pathogens are extremely resistant to cleansers and disinfectants [18].

Coagulase-positive staphylococci, including *S. aureus*, are a widespread group of microorganisms. *S. aureus* is responsible for a broad range of illnesses, including food poisoning characterized by intoxication [19]. Various factors, such as ingredients, production environment, and human transmission, can all contribute to their presence in food products. Contamination of raw milk with *S. aureus* may result from inflammation of the udder (mastitis) or may be caused by a failure to observe appropriate hygienic conditions during milking [19,20]. Usually, staphylococcal poisoning arises from the consumption of food containing enterotoxins. Staphylococcal enterotoxins (SEs) intoxication is characterized by a short incubation period of 0.5 to 8 h. Intoxication is characterized by, among other things, nausea, vomiting, abdominal pain, and diarrhea [7]. The five main enterotoxins produced by staphylococci (SEA, SEB, SEC, SED, and SEE) are responsible for 95% of these food poisoning cases. Staphylococcal enterotoxins are highly stable molecules, have high solubility in water and salt solutions, and are stable at pH ranging from 2 to 12 [21,22]. They exhibit resistance to heat treatment (they are not completely destroyed, e.g., during pasteurization for 15 s at 72 °C) and proteolytic enzymes, so they remain active in the digestive tract after contaminated food is consumed.

The aim of the study was to assess the microbiological risks associated with pathogenic bacteria (*Salmonella* spp., *L. monocytogenes*, coagulase-positive staphylococci) and toxins (staphylococcal enterotoxins) in Koryciński cheeses collected in 2021 and 2022 at retail. This is dictated by the increasing popularity of traditional and regional food in Poland, as well as recognizing the potential health effects of consuming unpasteurized dairy products.

## 2. Materials and Methods

### 2.1. Food Samples

The research material consisted of Koryciński cheeses sampled in 2021–2022 from grocery shops and markets located in Warsaw. A total of 45 cheese samples from various parties were taken from different producers, of which nine were natural, cream, and smoked cheeses, and 36 were cheese samples with different additives. Among the cheeses covered by the study, 18 cheese samples were purchased at various markets, and 27 were purchased at supermarkets. The distribution of samples from producers was as follows: market place 1–4 samples, market place 2–5 samples, market place 3–9, supermarket 1–18 samples, supermarket 2–5 samples, supermarket 3–2 samples, and supermarket 4–2 samples. The cheese samples were randomly selected from cheese blocks available for sale. A total of 150 g of each type of cheese was sampled. The cheese samples were transported to the laboratory in containers to prevent contamination in a portable refrigerator at 4 °C. The samples were stored at 3 ± 2 °C until the start of the tests [23].

The test samples were prepared in accordance with EN ISO 6887-5:2020-10 [24] and tested as follows:

- Detection of *Salmonella* spp., according to EN ISO 6579-1:2017-04+A1:2020 [25], using the XLD and Hektoen media (Biomerieux, Craponne, France), biochemical and serological confirmation using API tests (Biomerieux, France), and OM, HM, and Vi sera (Immunolab, Gdańsk, Poland).
- Enumeration of the *L. monocytogenes*, according to EN ISO 11290-2:2017-07 [26] using the ALOA medium (Biomerieux, France).
- Enumeration of the coagulase-positive staphylococci (*S. aureus* and other species) [27], according to EN ISO 6888-2:2001+A1:2004 [27] and the new edition of this standard EN ISO 6888-2:2022-03 [28], using an agar medium with rabbit plasma fibrinogen (Braid Parker+RPF, Biomerieux, France);
- Detection of staphylococcal enterotoxins in food when the coagulase-positive staphylococci count in a sample is more than 10^5^ cfu/g, according to EN ISO 19020:2017-08 [29], by an immunoenzymatic method using Vidas SET2 (Biomerieux, France).

The samples were tested using methods within the laboratory’s scope of accreditation.

### 2.2. Statistical Analysis

The statistical analysis was performed using STATISTICA 8.0 (Stat Soft, Kraków, Poland). The Mann–Whitney U test was used to find differences between the analyzed groups (i.e., natural, cream, and smoked cheeses and cheese samples with different additives). A statistical significance level was adopted for *p* ≤ 0.05.

## 3. Results and Discussion

The results of the tests carried out for *Salmonella* spp. and the *L. monocytogenes* count, the coagulase-positive staphylococci count, including *S. aureus*, and *S. aureus* toxins are shown in Table 1.

No *Salmonella* spp. was detected in 25 g of any of the 45 cheese samples tested. Comparable results were obtained by Brooks et al. [30], who, when assessing the microbiological safety of 41 cheeses made from raw milk, did not detect the presence of *Salmonella* spp. In 25 g of the samples tested. Also, Menendez et al. [31] did not detect the presence of *Salmonella* spp. in any of the 24 samples of “Tetilla” cheese made from raw cow’s milk. According to data published by EFSA on food safety monitoring in Member States in 2021, it appears that among the 0.12% of cheese samples out of 15,422 tested samples in which *Salmonella* spp. were found, there was no sample of cheese made from cow’s milk not subject to temperature treatment or subject to low-temperature heat treatment [12]. In 2021, the presence of *Salmonella* in a brie vegan alternative cheese was the cause of a salmonellosis outbreak in the United States, in which 20 people suffered poisoning, five of whom were hospitalized [32].

Based on the quantification of *L. monocytogenes*, it was found that the limit of 100 cfu/g was exceeded for four cheese samples tested. One of the cheeses (cheese with basil added) had elevated levels of *L. monocytogenes* contamination at 2.4 × 10^4^ cfu/g. For the sample of Koryciński cheese with cranberries, the estimated count of *L. monocytogenes* was 4.6 × 10^2^ cfu/g. In a further two cheese samples, microorganisms were present at less than 4 × 10^2^ cfu/g. In the remaining 41 samples (91.1% of all samples analyzed), the *L. monocytogenes* count was <100 cfu/g, i.e., in line with the requirements of Regulation 2073/2005 [8]. In a study conducted in Sweden by Loncarevic et al. [33], the quality of 333 cheese samples was determined, including those made from raw milk (31 samples) and pasteurized milk (302 samples). The tests detected *L. monocytogenes* in 20 out of 333 samples, ranging from 10^2^ to 10^5^ cfu/g. For cheeses made from raw milk, *L. monocytogenes* was found in 13 (42%) out of 31 cheese samples. However, in cheeses made from pasteurized milk, *L. monocytogenes* was found in 7 out of 302 samples analyzed, which accounts for only 2% of the samples in this category [33]. In the study by Pyz-Łukasik et al. [34], a total of 370 samples of artisanal raw milk cheeses were analyzed for the presence of *L. monocytogenes*. The cheese samples were collected from producers before they were put on the market. The pathogen was detected in 23 cheese samples, accounting for 6.2% of all samples tested. The research was conducted between 2014 and 2018 in southern Poland. Similar contamination of cheeses with *L monoctogenes* was found in a 2021 study in Egypt [35]. *L. monocytogenes* was detected in 2 out of 30 cheese samples, which constituted 6.66% of those tested. In research conducted in Duhok province, Iraq, of the 50 tested soft cheese samples, 3% were presumptively positive for *Listeria* spp., while 1 was confirmed as *L. monocytogenes* [36]. Cheese is a frequent vehicle for outbreaks involving *L. monocytogenes*. In 2021, *L. monocytogenes* present in fresh “queso fresco” cheese caused illness in 13 people, with 12 requiring hospitalization and one person dying [37]. In 2022, brie cheeses where *L. monocytogenes* were detected were the cause of a listeriosis outbreak in which five people needed hospitalization [38].

In the present study, *S. aureus* was detected in 31 out of 45 cheese samples tested, representing as much as 68.9% of the cheeses tested. These cheeses were made by different producers. The test results of samples taken from different production stages of artisanal cheeses made from unpasteurized milk conducted by Gajewska et al. [39] indicate coagulase-positive staphylococcal contamination in 55.6% of the samples tested.

Cheese tests carried out found contamination levels of samples with these pathogens ranging from 5.5 × 10^1^ cfu/g to 7.6 × 10^6^ cfu/g. The statistical analysis conducted revealed no statistically significant differences in the count of coagulase-positive staphylococci among the natural, cream, and smoked cheese samples when compared to cheeses with additives (Z = 0.20; *p* = 0.8397).

The mean count of coagulase-positive staphylococci in positive samples was 4.0 × 10^5^ cfu/g. In 25% of cheese samples tested, the level of *S. aureus* was similar to the values obtained in the analysis of raw milk cheeses presented by Wszołek et al. [40] (1.1 × 10^4^ cfu/g–3.1 × 10^4^ cfu/g).

In as many as 15 cheese samples, the level of *S. aureus* contamination was above 10^5^ cfu/g, and, therefore, the presence of staphylococcal enterotoxins was determined in these samples. Staphylococcal enterotoxins were found in one of the fifteen samples. Gajewska et al. [39] showed that artisanal cheese production could potentially lead to contamination of these products with multidrug-resistant and virulent strains of *S. aureus*. Martinez et al. [7] have demonstrated the presence of the gene fragments *sea*, *seb*, *sec*, *sed*, and *see* of enterotoxins in 68.8% of 77 *S. aureus* isolates derived from fresh artisanal cheese in Cuba, demonstrating the potential ability of *S. aureus* strains isolated from cheese to produce enterotoxins. In EFSA “One Health” reports, cheeses have been the leading food category most frequently linked to staphylococcal poisoning for many years [11]. Cheeses made from unpasteurized milk were also the source of food poisoning outbreaks involving *S. aureus* in 2009 in France. During this period, six local outbreaks caused by staphylococcal enterotoxins found in cheeses made from unpasteurized milk were reported. This serves as a confirmation that contamination of ingredient/products with staphylococci is a serious problem in the production of cheeses from raw milk [5].

**Figure 1:**
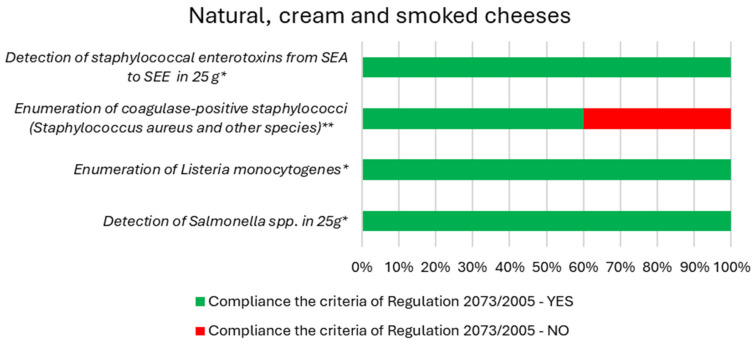
Results of compliance with the requirements of the microbiological criteria of Regulation. 2073/2005 for natural, cream, and smoked cheeses. * Food safety criteria. ** Process hygiene criteria.

**Figure 2:**
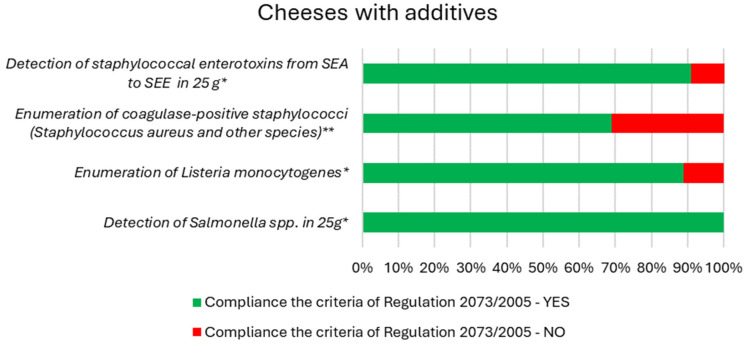
Results of compliance with the requirements of the microbiological criteria of Regulation. 2073/2005 for cheeses with additives. * Food safety criteria. ** Process hygiene criteria.

Figure 1 and Figure 2 present the results of compliance with the requirements of the microbiological criteria of Regulation. 2073/2005 for natural cream and smoked cheeses, as well as for cheeses with various additives, respectively. Tests carried out showed the presence of pathogenic bacteria, both in natural cheeses and in cheeses with additives. For cheeses with additives, more samples exceeding the limit for coagulase-positive staphylococci were observed compared to cream, natural, and smoked cheeses. However, taking into account the number of samples tested in these two different groups of cheeses, the percentage of samples that did not meet the hygienic criteria was higher for cream, natural, and smoked cheeses (40%) compared to cheeses with additives (31%). Furthermore, enterotoxin was found only in the cheese sample with additives. No *Salmonella* spp. was detected in either cheese with additives or natural cheese. All cheese samples complied with food safety criteria for *Salmonella* spp. However, *L. monocytogenes* was found in cheese samples with additives, therefore, 11% of the samples did not meet the food safety criteria. Najgebauer-Lejko et al. [41] evaluated the effect of milk from different sources and the addition of wild garlic on selected properties of soft rennet cheeses and found no effect of this addition on the number of bacteria, yeasts, and molds in the product.

A common trend is to raise public awareness of environmental issues and people’s attachment to local values. This is resulting in increased support for domestic and local agricultural production. It should also be noted that one of the primary criteria for consumer food choice is its safety. Evidence of the discussion regarding the compromise between the traditional method of production and the introduction of changes resulting from consumer expectations (the use of herbs and other spices) and consumer expectations regarding product safety and allowing the production of Korycin cheese from pasteurized milk. The announcement of the Minister of Agriculture and Rural Development dated 7 November 2022 on the application for approval of changes to the specification of “Ser korycinski swojski” [42], registered as a protected geographical indication, was cited as the most significant risk for cheeses made from unpasteurized raw milk with poor microbiological quality. It also shows that the introduction of the milk pasteurization process allows for the maintenance the expected sanitary standards for products. In connection with the above proposal, the European Commission adopted Commission Implementing Regulation (EU) 2024/326 of 11 January 2024 approving a Union amendment to the product specification for the protected geographical indication (‘Ser koryciński wojski’) [43].

In conclusion, this study has provided valuable insights into the microbiological risks associated with Koryciński cheeses available for retail. *Salmonella* spp. Was not found in any of the cheese samples tested, indicating a low risk of *Salmonella* spp. Occurrence in this type of food. However, quantification of *L. monocytogenes* showed that four samples exceeded the limit of 100 cfu/g, with one sample showing particularly high levels of contamination. This highlights the need for continuous monitoring and compliance with safety regulations to reduce the risk of listeriosis outbreaks, as exemplified by previous incidents involving *L. monocytogenes* contamination in cheese products.

Additionally, *S. aureus* was detected in the majority (68.9%) of the cheese samples tested, indicating widespread contamination across producers. The presence of coagulase-positive staphylococci in cheese samples highlights the importance of thorough hygiene practices throughout the production process. Moreover, the detection of staphylococcal enterotoxins in one sample emphasizes the potential health risks posed by contaminated cheeses.

Our research showed that slightly more samples that did not meet the criteria in at least one criterion were taken from the marketplace (44%), compared to the supermarket (37%).

The contamination observed in the tested cheese samples could potentially be attributed to inadequate hygienic conditions during both production and distribution, including staff hygiene practices. These findings highlight the complexity of microbiological risks in cheese production and underscore the importance of stringent quality control measures to ensure consumer safety.

## Figures and Tables

**Table 1:** Koryciński cheese test results.

Samples	Type of Koryciński Cheese	Shopping Location S-Supermarket M-Market Place	Detection of *Salmonella* spp. in 25 g	Enumeration of *Listeria monocytogenes* (cfu/g)	Enumeration of Coagulase-Positive Staphylococci (*Staphylococcus aureus* and Other Species)	Detection of Staphylococcal Enterotoxins from SEA to SEE in 25 g
1	with dried tomatoes and olives	S1	not detected	<100	7.6 × 10^4^	
2	with fenugreek	S1	not detected	<100	<10	
3	with basil	S1	not detected	2.4 × 10^4^	Estimated number of microorganisms 5.5 × 10^1^	
4	with cranberries	S1	not detected	Estimated number of microorganisms 4.6 × 10^2^	<10	
5	with nuts	S1	not detected	<100	1.2 × 10^5^	detected
6	with wild garlic	S1	not detected	<100	<10	
7	with black cumin	S1	not detected	<100	1.2 × 10^5^	not detected
8	natural	S2	not detected	<100	<10	
9	with black cumin	S2	not detected	<100	<10	
10	natural	S2	not detected	<100	<10	
11	natural	S1	not detected	<100	1.4 × 10^5^	not detected
12	with basil	S1	not detected	<100	<10	
13	with cranberries	S1	not detected	<100	1.6 × 10^5^	not detected
14	with wild garlic	S1	not detected	<100	<10	
15	with nuts	S1	not detected	<100	2.1 × 10^4^	
16	with black cumin	S1	not detected	<100	<10	
17	with fenugreek	S1	not detected	<100	<10	
18	with black cumin	S2	not detected	<100	3.4 × 10^5^	not detected
19	natural	S2	not detected	<100	2.0 × 10^4^	
20	smoked	S3	not detected	<100	1.1 × 10^5^	not detected
21	with herbs	S3	not detected	<100	3.7 × 10^5^	not detected
22	natural	M1	not detected	<100	5.6 × 10^4^	
23	with tomatoes and garlic	M1	not detected	<100	1.8 × 10^5^	not detected
24	with fenugreek	M1	not detected	<100	7.4 × 10^4^	
25	with tomatoes	M1	not detected	<100	6.8 × 10^4^	
26	natural	S1	not detected	<100	4.0 × 10^3^	
27	with Provençal herbs	S1	not detected	<100	<10	
28	with fenugreek	S1	not detected	<100	4.2 × 10^4^	
29	with tomatoes and chives	S1	not detected	<100	2.0 × 10^4^	
30	with cranberries	M2	not detected	<100	3.5 × 10^5^	not detected
31	with pistachios	M2	not detected	<100	2.5 × 10^5^	not detected
32	with fenugreek	M2	not detected	Microorganisms are present. but in numbers less than 4 × 10^2^ per gram	4.1 × 10^3^	
33	with basil	M2	not detected	<100	3.8 × 10^3^	
34	with wild garlic	M2	not detected	Microorganisms are present. but in numbers less than 4 × 10^2^ per gram	3.4 × 10^5^	not detected
35	natural	M3	not detected	<100	5.1 × 10^5^	not detected
36	with black cumin	M3	not detected	<100	2.1 × 10^3^	
37	cream	M3	not detected	<100	2.2 × 10^5^	not detected
38	with cranberries	M3	not detected	<100	<10	
39	with black cumin	M3	not detected	<100	3.4 × 10^4^	
40	natural	M3	not detected	<100	<10	
41	with black cumin	M3	not detected	<100	9.0 × 10^5^	not detected
42	with nuts	M3	not detected	<100	<10	
43	with wild garlic	M3	not detected	<100	7.1 × 10^4^	
44	with cranberries	S4	not detected	<100	2.6 × 10^3^	
45	with dried tomatoes	S4	not detected	<100	7.6 × 10^6^	not detected

## Data Availability

The original contributions presented in the study are included in the article, further inquiries can be directed to the corresponding author.
